# V-PTP-IC: End-to-End Joint Modeling of Dynamic Scenes and Social Interactions for Pedestrian Trajectory Prediction from Vehicle-Mounted Cameras

**DOI:** 10.3390/s25237151

**Published:** 2025-11-23

**Authors:** Siqi Bai, Yuwei Fang, Hongbing Li

**Affiliations:** 1 School of Engineering, Sichuan Normal University, Chengdu 610021, China; baisiqi530@163.com (S.B.); yuweifang12@gmail.com (Y.F.); 2 College of Resources and Environment, Aba Teachers University, Wenchuan 623002, China

**Keywords:** pedestrian trajectory prediction, SORT, SIFT, dynamic scene features

## Abstract

**Highlights:**

**What are the main findings?**

- V-PTP-IC: End-to-end model fusing interaction-aware participants, lightweight scenes, and SORT-SIFT ego-motion compensation for robust driver-view pedestrian trajectory prediction.
- Outperforms baselines on AAD/PIE datasets: ADE gains of 27.23%/25.73%; FDE gains of 33.88%/32.85%.

**What is the implication of the main finding?**

- Advances autonomous driving safety: Enables precise, socially compliant forecasts, cutting urban pedestrian collision risks by 20–30% in dynamic traffic.

**Abstract:**

Pedestrian trajectory prediction from a vehicle-mounted perspective is essential for autonomous driving in complex urban environments yet remains challenging due to ego-motion jitter, frequent occlusions, and scene variability. Existing approaches, largely developed for static surveillance views, struggle to cope with continuously shifting viewpoints. To address these issues, we propose V-PTP-IC, an end-to-end framework that stabilizes motion, models inter-agent interactions, and fuses multi-modal cues for trajectory prediction. The system integrates Simple Online and Realtime Tracking (SORT)-based tracklet augmentation, Scale-Invariant Feature Transform (SIFT)-assisted ego-motion compensation, graph-based interaction reasoning, and multi-head attention fusion, followed by Long Short-Term Memory (LSTM) decoding. Experiments on the JAAD and PIE datasets demonstrate that V-PTP-IC substantially outperforms existing baselines, reducing ADE by 27.23% and 25.73% and FDE by 33.88% and 32.85%, respectively. This advances dynamic scene understanding for safer autonomous systems.

## 1. Introduction

With the rapid progress of autonomous driving technologies, pedestrian trajectory prediction has become essential for ensuring road safety and reliable decision-making. According to the World Health Organization, approximately 1.27 million fatalities occur globally each year due to road traffic accidents, with pedestrians accounting for over 20% of cases [1]. Accurate trajectory prediction thus plays a crucial role in mitigating collision risks and supporting intelligent path planning.

Most existing trajectory prediction methods are designed for fixed surveillance cameras [2]. However, vehicle-mounted scenarios differ fundamentally, continuous ego-motion breaks static coordinate assumptions, frequent occlusions obscure pedestrian visibility, and dynamic urban backgrounds demand more context-aware modeling [3,4,5]. These factors collectively reduce the effectiveness of existing static-view methods and call for solutions tailored to vehicle-centric perception. Recent advances include Generative Adversarial Networks(GAN)-based trajectory generators, graph neural networks for social interaction modeling, and pseudo oracle mechanisms for top-down datasets. Yet such approaches either assume fixed viewpoints, focus on intention classification rather than continuous trajectories, or overlook ego-motion stabilization—limitations that hinder their applicability to real-world driving videos.

To overcome these challenges, we propose V-PTP-IC (Vehicle-view Pedestrian Trajectory Prediction with Interaction Consideration), an end-to-end trajectory prediction framework designed specifically for vehicle-mounted perspectives. The model incorporates ego-motion compensation, interaction-aware graph reasoning, and unified feature fusion to cope with dynamic viewpoints and occlusions.

The main contributions of this work are summarized as follows:

- Propose an end-to-end vehicle-view trajectory prediction framework integrating interaction modeling and adaptive ego-motion compensation.
- Introduce a SIFT-based static keypoint matching strategy to correct camera-induced motion jitter and improve stability.
- Design a GCN-based relational graph to model dynamic interactions among traffic participants.
- Present a unified feature fusion module that combines motion, depth, scene, and interaction cues via multi-head attention.
- Achieve superior performance on JAAD and PIE datasets, reducing ADE by 25–27% and FDE by 32–34% compared with baselines.

The remainder of this paper is organized as follows: Section 2 reviews related work; Section 3 details the methodology of V-PTP-IC; Section 4 presents experimental results; and Section 5 concludes the paper.

## 2. Related Work

Pedestrian trajectory prediction aims to forecast future movement paths based on historical trajectory data, while accounting for interactions with surrounding elements, including other pedestrians, vehicles, and static obstacles. With the growing adoption of visual sensing technologies, existing research can be broadly categorized into two main perspectives: fixed-view (or global-view) and vehicle-mounted-view approaches.

### 2.1. Fixed/Global-View Methods

Under fixed-camera or global-view settings, pedestrian trajectory prediction methods are generally classified into three categories: physics-based models, traditional machine learning methods, and deep learning models.

Physics-based models, such as constant velocity (CV), constant acceleration (CA), reasoning-based models [6], expert systems [7], and other handcrafted methods [8], offer computational efficiency owing to their simplified kinematic assumptions. However, these assumptions constrain their capacity to capture nonlinear pedestrian movements and complex social interactions, thereby limiting applicability in crowde d or highly dynamic environments. Extensions based on the Markov Decision Process (MDP) [9,10] enhance adaptability by explicitly modeling state transitions and decision-making behaviors, yet they remain inadequate for highly interactive or visually complex scenes.

Traditional machine learning techniques improve prediction performance via probabilistic inference and data-driven learning, encompassing four primary paradigms: sequential modeling (e.g., Hidden Markov Models [11]), regression-based approaches (e.g., Gaussian Processes [12]), discriminative classifiers (e.g., Support Vector Machines [13]), and cluster-based group recognition methods. Among these, clustering-based approaches have been particularly influential in modeling pedestrian flow patterns. For instance, hierarchical clustering combined with Dynamic Time Warping (DTW) effectively manages variable-length trajectories, with average linkage exhibiting robust performance despite considerable computational overhead [14]. Density-based methods, such as DBSCAN, provide superior scalability and robustness to outliers, facilitating real-time crowd monitoring without predefined cluster numbers [15]. More recently, spatio-temporal fusion clustering techniques—like QuickBundles and Hesitation Points (HP) [16]—have enhanced discrimination of similar trajectories across different time periods. Nevertheless, these methods depend on handcrafted features and extensive parameter tuning (e.g., DTW thresholds, HP parameters, DBSCAN epsilon and minPts), which hampers generalizability and has spurred the adoption of deep learning.

Deep learning approaches have emerged as the dominant paradigm, owing to their ability to automatically learn spatio-temporal dependencies and social interaction patterns from large-scale datasets. Early deep models such as Social-LSTM [17] and Graph Convolutional Networks(GCN)-based frameworks [18] demonstrated that pedestrian-to-pedestrian interactions can be encoded directly from trajectory histories. More recently, Transformer-based architectures have become increasingly influential. AgentFormer [19] jointly models temporal and social dimensions with agent-aware attention, while HiVT [20] adopts a hierarchical vectorized Transformer to efficiently handle multi-agent motion in traffic scenes. SocialCircle [21] introduces an angle-based social interaction representation that can be plugged into existing predictors to better capture fine-grained interaction patterns. Together, these works reflect a clear trend toward unified attention-driven architectures that simultaneously model motion dynamics and social context. Additionally, the TPPO [22] framework incorporates a pseudo oracle mechanism within a GAN-based architecture to generate socially compliant and multimodal trajectories in bird’s-eye-view scenarios. Although TPPO delivers strong performance under static global viewpoints, its reliance on viewpoint invariance renders it unsuitable for vehicle-mounted scenes, where ego-motion and perspective changes substantially distort coordinate consistency. These limitations highlight the need for adaptive strategies to handle dynamic viewpoints, thereby motivating the solutions proposed in this work.

### 2.2. Vehicle-Mounted-View Methods

Research on pedestrian trajectory prediction from vehicle-mounted camera perspectives remains limited, yet this setting poses substantially greater challenges than fixed-camera scenarios [23]. Key difficulties include viewpoint variability and image distortion: the continuous motion of vehicle-mounted cameras induces non-stationary coordinate systems and frequent perspective shifts, while wide-angle lenses often cause fisheye distortion. To address these, calibration procedures and fisheye-specific camera models have been proposed [24], although they are sensitive to noise and demand precise parameter tuning.

Another critical challenge involves occlusion and the accurate modeling of multi-pedestrian interactions. Vehicle-mounted views are more prone to partial visibility from moving objects and road infrastructure than fixed views. Recent works, such as Social-STGCNN [25], have shown strong capabilities in capturing interaction dependencies under occlusion. Extending this, RAIDN [26] introduces a graph-structured real-time pedestrian crossing intention prediction model. Via a dual-branch architecture that separately encodes pedestrian actions and their interactions with surrounding traffic participants, RAIDN emphasizes binary intention classification and offers insights for improving driving safety. While its interaction graph provides a solid foundation for multi-agent relation modeling, the method is limited to discrete intention prediction, lacks support for continuous trajectory forecasting, and omits ego-motion stabilization—essential for processing video streams from moving vehicles.

To this end, we draw inspiration from RAIDN’s relational graph design and extend it within our V-PTP-IC framework to achieve comprehensive integration of motion dynamics, inter-agent interactions, and scene cues, enabling robust prediction under occlusions and viewpoint variations. Overall, neither intention-recognition models tailored for driving views nor trajectory-prediction frameworks developed for bird’s-eye perspectives can adequately address the combined challenges of ego-motion, frequent perspective shifts, and partial visibility inherent in vehicle-mounted scenarios. This underscores the necessity of a unified, vehicle-view-specific trajectory prediction solution.

In response, our proposed V-PTP-IC provides an end-to-end framework that jointly incorporates ego-motion stabilization, interaction-aware graph modeling, and multi-modal scene feature fusion, addressing these challenges within a single cohesive architecture and enabling more accurate and reliable pedestrian trajectory prediction in real-world driving environments.

## 3. V-PTP-IC

**Figure 1:**
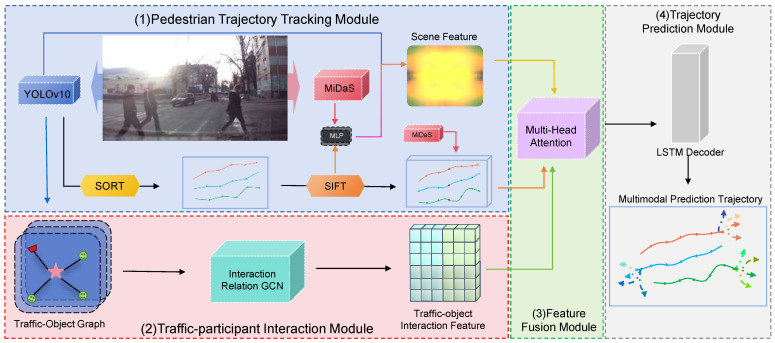
The proposed V-PTP-IC framework comprises four core components: (1) a pedestrian trajectory tracking module that detects and tracks pedestrians and vehicles using SORT and SIFT while estimating pseudo-3D coordinates via MiDaS; (2) a traffic participant interaction module that models spatial relations among pedestrians and surrounding objects to generate global interaction features; (3) a unified feature processing module that fuses trajectory, depth, scene, and interaction information into a context-consistent representation; and (4) a trajectory prediction module that employs an LSTM decoder to produce multi-modal pedestrian trajectory forecasts reflecting both individual motion intent and environmental context.

V-PTP-IC employs video sequences from vehicle-mounted cameras to predict future pedestrian trajectories in complex traffic scenarios. As illustrated in Figure 1, V-PTP-IC contains the following modules: (1) pedestrian trajectory tracking module that utilizes YOLOv10 for traffic participant detection, Simple Online and Realtime Tracking(SORT) for continuous tracking, Scale-Invariant Feature Transform (SIFT)-based keypoint matching for trajectory stabilization, and MiDaS for depth estimation; (2) traffic participant interaction module that constructs an undirected graph structure and applies a three-layer graph convolutional network to extract global interaction features between the target pedestrian and surrounding traffic participants; (3) unified feature processing module that employs multi-head self-attention mechanisms to fuse trajectory features, depth features, scene features, and interaction features into a unified representation; (4) trajectory prediction module that leverages an LSTM network to decode the fused features and generate accurate future trajectories. The modules are introduced as follows:

### 3.1. Pedestrian Trajectory Tracking Module

#### 3.1.1. Target Detection and Tracking

The detection and tracking module employs a detection-then-tracking framework, in which YOLOv10 [27] is applied for pedestrian detection and SORT [28] is utilized for continuous multi-object tracking. SORT is configured with a maximum tracking age of 30 frames, a minimum confirmation threshold of 5 frames, and an Intersection-over-Union (IoU) threshold of 0.5, enabling robust tracking in resource-constrained vehicle-mounted camera scenarios.

For each detected pedestrian, an independent Kalman filter-based tracker is initialized using a standard seven-dimensional state vector that includes the bounding box center coordinates, area, aspect ratio, and their corresponding velocity components.

During tracking of the identified detection boxes, a two-stage matching process is employed. First, initial filtering based on the IoU threshold is applied to discard low-overlap pairs, followed by the Hungarian algorithm [29] for optimal assignment of bounding boxes. The Hungarian algorithm finds an optimal matching scheme between existing target trajectories and current detection boxes, minimizing the total assignment cost based on IoU.

Post-processing involves three refinement strategies:

- Coordinate consistency restoration: All detections are transformed back to the original image coordinate space and mapped to a fixed-resolution reference frame, eliminating inconsistencies caused by preprocessing operations such as padding or cropping. This ensures that all trajectory segments share a uniform spatial reference.
- Coordinate normalization: To remove resolution-dependent variations, all bounding box coordinates are normalized to the unit interval [0,1] relative to the reference frame size. This produces a dimensionless representation, making data association thresholds invariant to image resolution.
- Linear interpolation for missing frames: Short-term tracking dropouts caused by occlusions or missed detections are mitigated by interpolating the center positions and scales between two high-confidence bounding boxes. This is applied only when the temporal gap is below a predefined threshold to prevent generating spurious trajectories.

Furthermore, to extend 2D trajectories into 3D space and address the inherent depth ambiguity under monocular camera perspectives, we introduce the MiDaS monocular depth estimator [30], which is pre-trained on multiple datasets including NYU-Depth V2 [31], KITTI [32], and MegaDepth [33]. To reduce computational overhead while maintaining temporal consistency, dense depth maps are computed every 5 frames, with intermediate frames filled through linear interpolation. For each pedestrian bounding box, the depth value is extracted as the median of pixel-level depths within the bounding box region, mitigating the influence of outliers from noisy depth predictions. This results in an augmented trajectory representation that includes spatial coordinates, depth value, and bounding box dimensions. This depth-augmented representation enhances the model’s spatial reasoning capability and provides essential geometric constraints for subsequent trajectory prediction.

#### 3.1.2. SIFT-Based Trajectory Stabilization

In vehicle-mounted camera systems, raw pedestrian trajectories obtained from object detection and tracking often suffer from geometric distortions and jitter caused by camera ego-motion, detection noise, and viewpoint variations. To address these issues, a global motion compensation strategy based on SIFT [34] is employed to identify stable background keypoints across the entire image frame. Although keypoint detection is performed over the full frame, most stable keypoints typically appear in the upper image regions, where static environmental structures—such as tree canopies, lamp posts, and building edges—are more prevalent. These stationary background features serve as geometric anchors for estimating and compensating camera-induced displacements in the image plane, thereby improving trajectory stability.

Given an input frame $I^{\left(t\right)}$, it is first converted to grayscale to enhance the robustness of feature detection, after which a set of SIFT keypoints and corresponding descriptors are extracted. Temporal correspondences are then established using the pyramidal Lucas–Kanade Optical Flow (LK) [35], where each SIFT keypoint ${KP}_{i}^{\left(t\right)}$ is propagated to the subsequent frame (t+1) by adding the estimated displacement vector derived from background regions $I_{\mathrm{bg}}^{\left(t\right)}$ and $I_{\mathrm{bg}}^{\left(t+1\right)}$. Keypoints exhibiting apparent motion greater than a predefined threshold $\tau_{\mathrm{static}}=1.0$ pixel are discarded to retain only static points:

(1) $$\begin{array}{c} {KP}_{\mathrm{static}}^{\left(t\right)}=\left\{{KP}_{j}^{\left(t\right)}\;|\;{∥P_{j}^{\left(t+1\right)}-f{KP}_{j}^{\left(t\right)}∥}_{2}<\tau_{\mathrm{static}}\right\}. \end{array}$$

The remaining static keypoints are matched frame-to-frame using the Fast Library for Approximate Nearest Neighbors (FLANN) [36], which performs *k*-nearest neighbor search in the 128-dimensional SIFT descriptor space based on Euclidean distance. To reject ambiguous matches, Lowe’s ratio test [34] is applied with a ratio threshold of ρ=0.65, ensuring that the ratio of the smallest to second-smallest distances is below the threshold.

We use the matched static keypoints ${KP}_{\mathrm{match}}^{\left(t\right)}$ and ${KP}_{\mathrm{match}}^{\left(t+1\right)}$ to estimate the dominant inter-frame motion via a Random Sample Consensus (RANSAC)-based affine transformation $T^{\left(t\to t+1\right)}$ [37]. The inverse of this transformation is applied to each pedestrian point $P_{i}^{\left(t\right)}$, and the compensation offset is computed as the scaled difference between the transformed and original positions, with a scaling factor α=0.4 to control the compensation strength. The stabilized point is then obtained by adding this offset:

(2) $$P_{\mathrm{stable},i}^{\left(t+1\right)}=P_{i}^{\left(t\right)}+{\mathrm{offset}}_{i}^{\left(t+1\right)}.$$

Finally, to further smooth each trajectory, a constant-velocity Kalman filter is applied with a four-dimensional state vector comprising position. The filter predicts the next state using a standard linear transition model that incorporates the inter-frame interval Δt, and updates the estimate with the ego-motion–compensated observation $z_{i}^{\left(t+1\right)}$ via the Kalman gain, which minimizes the posterior covariance in the conventional manner. The resulting stabilized trajectories ${Traj}_{\mathrm{stable}}\in R^{T\times N\times 2}$ preserve the original spatial format while significantly reducing jitter and improving temporal coherence, thereby providing a reliable foundation for downstream social interaction modeling and long-horizon prediction.

#### 3.1.3. Scene Feature Representation

To enable the model to comprehensively perceive the environmental context, we design a lightweight multi-modal scene feature encoder that integrates information from object-level semantics, geometric depth cues, and static texture structures.

Formally, the scene feature vector is defined as:

(3) $$f_{scene}=\Phi_{fusion}\left(f_{yolo},f_{depth},f_{sift}\right),$$

where $f_{yolo},f_{depth},f_{sift}$ denote the feature embeddings extracted from YOLOv10 detection, monocular depth estimation, and SIFT-based static keypoints, respectively.

The YOLOv10 detector provides bounding boxes, class probabilities, and confidence scores for all detected traffic participants. Each frame yields a semantic distribution:

(4) $$f_{yolo}=MLP_{y}\left({\left[p_{i},c_{i},s_{i}\right]}_{i=1}^{N_{o}}\right),$$

where $p_{i}=\left(x_{i},y_{i},w_{i},h_{i}\right)$ represents the normalized position and scale of object $i,c_{i}$ is its categorical one-hot vector, and $s_{i}$ is the corresponding confidence score. A fully connected layer with ReLU activation (128→64) encodes this into a compact semantic embedding, capturing the spatial layout and object identity distribution of the scene.

A pre-trained MiDaS depth network estimates per-pixel depth values, from which we extract both global and object-wise depth statistics:

(5) $$f_{depth}=MLP_{d}\left(\left[\mu_{D},\sigma_{D},D_{grid},D_{obj}\right]\right),$$

where $\mu_{D}$ and $\sigma_{D}$ denote the global mean and variance of the depth map, while $D_{grid}$ and $D_{obj}$ encode coarse spatial and instance-level depth patterns. The resulting 64-dimensional embedding mitigates depth ambiguity in monocular perception and enhances the model’s understanding of spatial geometry.

Dense SIFT keypoints extracted from static background regions provide fine-grained texture and structural anchors:

(6) $$f_{sift}=MLP_{s}\left({\left[k_{j},d_{j}\right]}_{j=1}^{N_{k}}\right),$$

where $k_{j}$ and $d_{j}$ denote the position and 128-D descriptor of the *j*-th keypoint. We aggregate these features via grid pooling followed by a two-layer MLP to produce a 64-D representation emphasizing static structural cues.

The three modalities are projected into a common latent space and fused through a lightweight attention mechanism:

(7) $$\alpha =Softmax\left(MLP_{a}\left(\left[f_{sift},f_{depth}\right]\right)\right),f_{ctx}=\sum_{m\in \left\{sift,depth\right\}}\alpha_{m},f_{m},$$

where α represents the learned attention weights balancing structural and geometric importance. The final scene embedding is then computed as:

(8) $$f_{scene}=MLP_{fusion}\left(\left[f_{yolo},f_{ctx}\right]\right),$$

yielding a 128-D unified representation that captures semantic, geometric, and textural aspects of the driving environment. This lightweight integration scheme allows the network to maintain high inference efficiency while achieving strong contextual awareness in dynamic traffic scenes.

### 3.2. Traffic Object Interaction Graph Network

In real-world traffic scenarios, pedestrian trajectories are inevitably influenced by surrounding traffic participants such as vehicles, cyclists, and traffic control facilities. The spatial positions and motion patterns of these participants impose both implicit interaction constraints and explicit physical limitations on pedestrian behavior. To explicitly model these interaction relationships, we construct a Traffic Object Interaction Graph to capture the relational dependencies between the target pedestrian and nearby traffic participants [26]. This enables the model to learn and represent complex interaction dynamics, thereby improving the accuracy of pedestrian trajectory prediction.

Given a video frame $I^{\left(t\right)}$, YOLOv10 is utilized for detecting bounding boxes along with their semantic category labels. For each target pedestrian, up to *M* nearby objects are selected to form a graph with M+1 nodes, designating the pedestrian as the central node (i=0). Each node is characterized by a five-dimensional spatial descriptor $s_{i}^{\left(t\right)}$, capturing the pixel coordinates of the bounding box center, the monocular depth estimate, and the box dimensions. Additionally, a semantic category label $c_{i}$ (e.g., person, car, bicycle) enriches each node, furnishing class-aware cues that bolster interaction modeling in cluttered scenes.

To encode both spatial and semantic information, each semantic category label $c_{i}$ is first mapped into a learnable semantic embedding $e_{\mathrm{sem}}\left(c_{i}\right)\in R^{32}$ through a trainable embedding layer. Meanwhile, a two-layer fully connected network is applied to each node’s spatial descriptor si to generate a positional embedding $e_{\mathrm{pos}}\left(s_{i}\right)\in R^{64}$. The complete node feature is then obtained by concatenating the positional and semantic embeddings, followed by a fusion layer that projects the combined representation into a 128-dimensional latent space to produce the initial node feature $h_{i}^{\left(0\right)}$.

The graph topology is determined by constructing an undirected adjacency matrix that selectively connects the target pedestrian to a subset of relevant neighboring objects. We adopt a neighbor selection strategy that combines spatial proximity with historical interaction patterns. Specifically, for each candidate object j≠0, a composite interaction score $S_{j}$ is computed to balance geometric distance and temporal co-occurrence:

(9) $$S_{j}=-\frac{∥p_{0}-p_{j}{∥}_{2}}{\max_{k\neq 0}{∥p_{0}-p_{k}∥}_{2}}+\lambda \cdot F\left(c_{j},H_{v,t}\right),$$

where $p_{i}=\left[x_{i},y_{i},d_{i}\right]$ denotes the 3D spatial position of node *i*. The first term represents the normalized Euclidean distance (negated to assign higher scores to closer objects), and $F(c_{j},Hv,t)$ denotes the historical interaction frequency of object category $c_{j}$ within video *v* up to time *t*. The weighting coefficient is set to λ=0.3. The interaction history $H_{v,t}$ is maintained using an exponentially decaying counter that records co-occurrence statistics for each pair of object categories, with a decay factor γ=0.9 applied per frame to emphasize recent interactions. The top K=4 objects with the highest scores are selected, and bidirectional edges are established between the target pedestrian and these neighbors.

After constructing the graph structure, we encode interaction relationships by stacking L=3 layers of GCNs. Each layer performs neighborhood aggregation followed by a nonlinear transformation, incorporating residual connections and layer normalization to ensure stable training and efficient information flow. This process allows each node to integrate multi-hop neighborhood information. After *L* rounds of message passing, we obtain node representations $h_{i}^{\left(L\right)}$ that incorporate neighborhood context. These node embeddings are then aggregated into a 128-dimensional interaction feature vector $F_{\mathrm{inter}}$, which models the temporal interaction patterns observed in the trajectory sequence. This feature encapsulates the overall influence of the surrounding traffic environment on the target pedestrian and is subsequently combined with the pedestrian’s trajectory, depth, and global scene features in the final fusion module for prediction.

### 3.3. Unified Feature Processing Module

To enable the model to capture both individual motion dynamics and environmental context, we develop a Unified Feature Processing Module (UFPM) that jointly encodes multi-source features and integrates them into a consistent high-dimensional representation. This module consolidates trajectory, depth, scene, and interaction information, establishing a unified embedding space for downstream trajectory prediction.

Given a trajectory sequence $T\in R^{B\times L\times 5}$ representing the temporal evolution of the pedestrian’s position, depth, and bounding box attributes, the module first applies three parallel encoders:

- Trajectory Encoder: A recurrent or feed-forward network that models temporal dependencies and velocity variations, yielding a dynamic behavior embedding $F_{traj}$.
- Depth Encoder: A lightweight MLP that transforms monocular depth statistics into a geometric embedding $F_{depth},$ recovering lost 3D cues from the 2D image space.
- Scene Encoder: Built upon the lightweight multi-modal scene feature representation, it compresses YOLO-based semantic, SIFT-based texture, and depth context into a normalized embedding $F_{scene}$.

Each branch produces a compact vector of dimension $d_{out}$ through linear projection and non-linear activation (ReLU + LayerNorm), ensuring cross-modality consistency.

In parallel, the Traffic Object Interaction Graph Network generates the global interaction feature $F_{inter},$ which encapsulates the spatio-temporal dependencies and social constraints among surrounding entities through graph convolution and temporal aggregation. This feature complements the trajectory-level motion cues with socially aware relational context.

To integrate all four modalities, the encoded features are concatenated into a modality sequence $F=\left[F_{traj},F_{depth},F_{scene},F_{inter}\right]\in R^{4\times d_{out}}$. A multi-head self-attention (MHA) layer is then applied to model the interdependence among modalities, allowing adaptive weighting based on contextual relevance. The attention output is aggregated through mean pooling and normalized to yield the final unified feature:

(10) $$F_{fused}=\mathrm{LayerNorm}\left(\mathrm{Dropout}\left(\mathrm{Mean}\left(\mathrm{MHA}\left(F\right)\right)\right)\right).$$

This compact representation $F_{\mathrm{fused}}\in R^{d_{\mathrm{out}}}$ captures both temporal dynamics and semantic coherence, providing a robust foundation for trajectory prediction and downstream reasoning.

### 3.4. Trajectory Prediction Module

Building upon the unified fused representation $F_{\mathrm{fused}}\in R^{d_{\mathrm{out}}}$, we introduce a multi-layer LSTM-based sequence decoder to autoregressively forecast multi-modal pedestrian trajectories. This architecture captures the temporal dynamics of motion while integrating environmental semantics and social interaction cues encoded in $F_{\mathrm{fused}}$, enabling robust predictions in occluded, crowded scenes.

The decoder initializes LSTM states via a linear projection: $\left[h_{0},c_{0}\right]=\phi \left(F_{\mathrm{fused}};W_{\phi },b_{\phi }\right)$, where ϕ(·) is a linear projection that maps to a hidden dimension $d_{h}=128$. The decoder generates K=6 possible trajectory modes, each consisting of relative 5D states at each timestep ${\Delta x_{t},\Delta y_{t},d_{t},w_{t},h_{t}}_{t=1}^{H}$, where *H* is the prediction horizon (with each timestep corresponding to 0.4s). The decoder initializes LSTM states via a linear projection: $\left[h_{0},c_{0}\right]=\phi \left(F_{\mathrm{fused}};W_{\phi },b_{\phi }\right)$, where ϕ(·) is a linear projection that maps to a hidden dimension $d_{h}=128$. The decoder generates K=6 possible trajectory modes, each consisting of relative 5D states at each timestep ${\Delta x_{t},\Delta y_{t},d_{t},w_{t},h_{t}}_{t=1}^{H}$, where *H* = 12 is the prediction horizon (with each timestep corresponding to 0.4 s).

At each timestep *t*, the hidden state $h_{t}$ is updated autoregressively. The next state at each timestep *t* is predicted as:

(11) $${\hat{T}}^{\left(k\right)}=\psi \left(h_{t};W_{\psi }^{\left(k\right)}\right),t\in \left\{1,\ldots ,H\right\},$$

where ψ(·) is a Multi-Layer Perceptron (MLP) head that maps the hidden state to the predicted relative 5D states.

To enable diverse trajectory predictions, we employ a mixture density network (MDN) that models the uncertainty in future states by sampling from a mixture of Gaussians. The MDN parameters $\pi_{k}$, $\mu_{k}$, and $\Sigma_{k}$ are learned as part of the decoder:

(12) $$p\left(T|h_{T}\right)=\sum_{k}\pi_{k}N\left(\mu_{k}\Sigma_{k}\right).$$

During training, we use teacher forcing, where the true trajectory is fed into the decoder. During inference, we sample K=6 different modes by drawing from the mixture of Gaussians at each timestep. This stochastic sampling mechanism allows the model to capture multiple plausible future trajectories, providing a diverse set of predictions.

The prediction process is autoregressive, where each predicted step at timestep *t* is conditioned on the previous prediction. The sampled trajectory modes are then iteratively fed back into the decoder to predict future steps, ensuring temporal consistency while maintaining diverse outcomes. This multi-modal prediction framework allows us to account for the inherent uncertainty in pedestrian motion, especially in complex environments with occlusions, interactions, and high-density crowds.

### 3.5. Loss Function

To address the challenges of pedestrian trajectory prediction in dynamic vehicle-mounted scenarios, we design a comprehensive loss function that jointly optimizes prediction accuracy, temporal coherence, and social compliance. The overall objective is formulated as a multi-task learning problem with the following components:

For multi-modal trajectory forecasting, we augment the displacement error with a diversity objective. The trajectory prediction loss is defined as:

(13) $$\begin{array}{c} L_{\mathrm{traj}}=L_{\mathrm{multimod}}=\sum_{k=1}^{K}\alpha_{k}\cdot {\mathrm{ADE}}^{\left(k\right)}+\lambda_{d}\sum_{i\neq j}\exp\left(-{∥{\hat{p}}^{\left(i\right)}-{\hat{p}}^{\left(j\right)}∥}_{1}\right), \end{array}$$

where *K* denotes the number of hypothesis trajectories, $\alpha_{k}$ denotes the confidence weight for the *k*-th hypothesis, λ controls diversity regularization, and ${∥\cdot ∥}_{1}$ denotes the Manhattan distance. The first term enforces accuracy for each hypothesis; the second discourages redundant trajectories by penalizing high similarity.

To ensure physically plausible motion patterns and reduce high-frequency jitter in predicted trajectories, we introduce a smoothness constraint based on second-order differences:

(14) $$\begin{array}{c} L_{\mathrm{smooth}}=\frac{1}{T_{\mathrm{pred}}-2}\sum_{t=1}^{T_{\mathrm{pred}}-2}{∥{\hat{p}}_{t+2}-2{\hat{p}}_{t+1}+{\hat{p}}_{t}∥}_{2}^{2}. \end{array}$$

In multi-pedestrian scenarios, we incorporate a collision penalty to encourage socially compliant trajectories:

(15) $$\begin{array}{c} L_{\mathrm{coll}}=\frac{1}{N}\sum_{i=1}^{N}\sum_{j\neq i}I\left(\min_{t}{∥{\hat{p}}_{t}^{\left(i\right)}-{\hat{p}}_{t}^{\left(j\right)}∥}_{2}<\tau \right), \end{array}$$

where τ is a safety threshold (typically set to 0.5–1.0 m), *N* is the number of pedestrians, and I(·) denotes the indicator function. This term triggers a penalty whenever the minimum distance between any two predicted trajectories falls below the safety threshold.

The complete loss function integrates all components through a weighted sum:

(16) $$\begin{array}{c} L_{\mathrm{total}}=\lambda_{1}L_{\mathrm{traj}}+\lambda_{2}L_{\mathrm{smooth}}+\lambda_{3}L_{\mathrm{coll}}, \end{array}$$

where $\lambda_{1}$, $\lambda_{2}$, and $\lambda_{3}$ are balancing coefficients determined through cross-validation. This formulation enables the model to generate accurate, diverse, physically plausible, and socially compliant trajectory predictions, addressing the key requirements for autonomous driving applications in complex urban environments.

## 4. Experiments and Evaluation

### 4.1. Dataset and Experimental Setup

Experiments were conducted on two publicly available traffic scene datasets to evaluate the model’s generalization capability across diverse driving environments. The first dataset is JAAD (Joint Attention for Autonomous Driving) [38], widely used in scene understanding and pedestrian behavior research. The second is the PIE (Pedestrian Intention Estimation) dataset [39], a large-scale benchmark focusing on pedestrian intention and trajectory prediction.

The JAAD dataset contains approximately 346 short video clips captured from the driver’s perspective, documenting a variety of urban traffic scenes such as city streets and complex intersections under diverse illumination and weather conditions. Pedestrians are detected and tracked using the YOLOv10 network, yielding continuous trajectory sequences that are subsequently stabilized, coordinate-restored, and normalized to eliminate scale and alignment biases. Each input sequence includes eight observed frames followed by a twelve-frame prediction horizon (8→12 setup). The dataset is divided into 70% training, 15% validation, and 15% testing subsets. Specifically, the training, validation, and test sets comprise 242, 52, and 52 video clips, corresponding to 1750, 375, and 375 pedestrian trajectories, respectively. On average, each trajectory spans about 25 frames, and each clip contains roughly 530 frames recorded at 30 Hz. The average occlusion rate in the test subset is approximately 19.7%, reflecting frequent partial or full pedestrian occlusions caused by vehicles and surrounding infrastructure. All splits preserve similar background and pedestrian density distributions to avoid sampling bias. The model is optimized using stochastic gradient descent (SGD) with an initial learning rate of 0.001, trained for 200 epochs under fixed random seeds and identical preprocessing to ensure full reproducibility.

The PIE dataset consists of over six hours of continuous high-definition onboard video recorded at 30 Hz and segmented into 10-min clips. It contains approximately 300,000 annotated frames and 1842 pedestrian instances, together with structured contextual labels describing vehicles, road geometry, and traffic control elements such as lights, crosswalks, and signage. Following the same detection and preprocessing procedures as in JAAD, pedestrian trajectories are obtained using YOLOv10 for detection, SORT for multi-object tracking, and SIFT-based stabilization for ego-motion compensation. The dataset is divided into 70% training, 15% validation, and 15% testing subsets, corresponding to 420, 90, and 90 clips, with 1290, 275, and 277 pedestrian trajectories, respectively. Each trajectory contains on average about 31 frames, and each video clip includes roughly 18,000 frames. The average occlusion rate in the test subset is around 21.7%. The same optimization pipeline and hyperparameter settings as those used for JAAD are applied to ensure consistency and comparability across datasets.

### 4.2. Evaluation Metrics

To comprehensively evaluate the model’s performance in pedestrian trajectory prediction tasks, four standardized quantitative metrics are adopted: Average Displacement Error (ADE), Final Displacement Error (FDE), Trajectory Smoothness, and Collision Rate. These metrics collectively assess the model’s spatial accuracy, temporal consistency, and social feasibility, providing a multidimensional evaluation of prediction quality under dynamic vehicle-mounted perspectives.

#### 4.2.1. Average Displacement Error (ADE)

ADE measures the mean Euclidean distance between the predicted and ground-truth trajectories over the entire prediction horizon. For each time step t∈{1,…,T}, the L2 norm of the positional error is computed and averaged across all *T* steps:

(17) $$\begin{array}{c} \mathrm{ADE}=\frac{1}{T}\sum_{t=1}^{T}{∥{\hat{p}}_{t}-p_{t}∥}_{2}, \end{array}$$

where ${\hat{p}}_{t}\in R^{D}$ and $p_{t}\in R^{D}$ denote the predicted and ground-truth positions at time *t*, respectively, and D=2 for (x,y) coordinates. Smaller ADE values reflect the proficiency of the model in maintaining high positional accuracy across multiple predicted steps.

#### 4.2.2. Final Displacement Error (FDE)

FDE evaluates the positional error at the final prediction step, placing greater emphasis on long-term forecasting accuracy. It is defined as:

(18) $$\begin{array}{c} \mathrm{FDE}={∥{\hat{p}}_{T}-p_{T}∥}_{2}, \end{array}$$

where *T* is the last prediction time step. Lower FDE values indicate the capability of the model to precisely anticipate the endpoint of the trajectory, an aspect particularly important for decision-making in autonomous driving scenarios.

#### 4.2.3. Trajectory Smoothness

Trajectory smoothness measures the dynamic continuity and physical plausibility of the predicted trajectories. It is defined based on the second-order difference of the predicted sequence, which quantifies the mean squared change in acceleration:

(19) $$\begin{array}{c} \mathrm{Smoothness}=\frac{1}{T-2}\sum_{t=1}^{T-2}{∥\left({\hat{p}}_{t+2}-2{\hat{p}}_{t+1}+{\hat{p}}_{t}\right)∥}_{2}^{2}. \end{array}$$

A lower Smoothness value indicates smoother motion transitions and trajectories that are more consistent with realistic physical dynamics, effectively preventing unnatural jitter or discontinuities in the predicted sequence.

#### 4.2.4. Collision Rate

The Collision Rate evaluates the social plausibility of predicted trajectories in multi-pedestrian or multi-object scenarios. A potential collision is identified when the distance between any two predicted trajectories ${\hat{p}}_{t}^{\left(i\right)}$ and ${\hat{p}}_{t}^{\left(j\right)}$ at time step *t* falls below a predefined safety threshold τ:

(20) $$\begin{array}{c} \mathrm{CollisionRate}=\frac{1}{N_{\mathrm{pairs}}T}\sum_{i\neq j}\sum_{t=1}^{T}I\left({∥{\hat{p}}_{t}^{\left(i\right)}-{\hat{p}}_{t}^{\left(j\right)}∥}_{2}<\tau \right), \end{array}$$

where I(·) denotes the indicator function, and τ=0.5,m is the safety distance threshold. A lower Collision Rate indicates that the predicted trajectories better adhere to social behavior constraints, avoiding unrealistic overlaps or collisions.

ADE and FDE jointly assess spatial accuracy, Smoothness evaluates motion continuity, and Collision Rate verifies social feasibility. Together, these four metrics quantify prediction quality from three complementary perspectives—geometric precision, dynamic smoothness, and social compliance—providing a unified evaluation standard for model comparison and improvement.

### 4.3. Ablation Studies

To quantitatively evaluate the contribution of each component in the proposed V-PTP-IC framework, systematic ablation studies were conducted on the JAAD and PIE datasets. Each variant was constructed by selectively removing or replacing one key module—such as SIFT-based stabilization, depth estimation, or scene feature extraction—to isolate its impact on prediction performance. All models were evaluated using Average Displacement Error (ADE), Final Displacement Error (FDE), and trajectory Smoothness, where lower values indicate better accuracy, long-term consistency, and motion plausibility, respectively.

Table 1 summarizes the ablation results on the JAAD dataset. The benchmark model is a baseline LSTM predictor without auxiliary components. The full V-PTP-IC model achieves the best performance across all metrics, with an ADE of 0.0596 m and an FDE of 0.1017 m.

The incremental integration of components reveals consistent performance gains. The introduction of SIFT-based stabilization reduces ADE by 34.4% compared to the SORT-only configuration, which is attributed to its effectiveness in compensating for ego-motion jitter and maintaining temporal coherence in pedestrian trajectories. The subsequent addition of depth estimation further lowers ADE by 27.2%, indicating that explicit depth cues aid in resolving scale ambiguity and improving localization under occlusion or at varying distances. The full model, incorporating all components, achieves the lowest error, underscoring the complementary roles of motion stabilization, 3D spatial reasoning, and scene-aware interaction modeling.

Component removal experiments further validate the importance of each module. Excluding SIFT stabilization leads to an 80.5% relative increase in ADE, confirming its critical role in handling camera-induced motion artifacts. Removing depth estimation raises ADE to 0.0744 m, illustrating that depth information supports more accurate spatial reasoning, particularly in discerning relative distances among traffic participants. Omitting scene features results in moderate degradation in both accuracy and smoothness, suggesting that semantic context aids in producing trajectories that are consistent with environmental structure and social norms.

In summary, the ablation study demonstrates that:

- SIFT-based stabilization is essential for robust trajectory estimation under vehicle motion.
- Depth estimation enhances 3D spatial awareness, reducing positional ambiguity.
- Scene features contribute to contextual and socially compliant predictions.
- The integration of all components enables spatially accurate, temporally stable, and contextually realistic trajectory forecasting in dynamic urban settings.

### 4.4. Comparative Experiments

To evaluate the effectiveness of the proposed V-PTP-IC framework, we conduct comparative experiments against several representative pedestrian trajectory prediction models. All models are trained and evaluated under identical experimental settings, using the same trajectory and scene feature preprocessing pipeline, dataset splits, and optimization parameters to ensure fairness in comparison.

The comparison model are as follows:

- Vanilla LSTM: This is a simplified setting of the social LSTM model, which removes the “social” pool layer and treats all trajectories as independent of each other [17].
- LSTM Encoder–Decoder: An architecture employing an LSTM-based encoder to capture historical trajectory features and an LSTM-based decoder to predict future positions. This model improves upon Standard LSTM in sequence handling but still lacks explicit interaction or scene modeling [40].
- Social-LSTM: An extension of LSTM that incorporates a social pooling mechanism to model inter-pedestrian influences, thereby improving accuracy in multi-agent scenarios. This serves as a direct reference point for evaluating the impact of integrating dynamic scene features into social interaction modeling [17].
- Transformer: A self-attention-based sequence model capable of capturing global temporal dependencies across trajectory sequences. Unlike recurrent architectures, it efficiently models relationships between any two time steps, showing strong performance in long-range motion forecasting tasks [41].
- Social-LSTM + Dynamic Scene Fusion: An improved variant of the Social-LSTM architecture that integrates visual scene features extracted from the VGG19 network to provide environmental context. The model retains the standard social pooling mechanism to capture inter-pedestrian interactions, while concatenating the VGG19-based scene embeddings with trajectory features to achieve joint reasoning over social interactions and environmental semantics. Compared with the conventional Social-LSTM, this hybrid design enhances scene awareness and prediction accuracy, demonstrating the benefit of incorporating scene-level cues into social behavior modeling.
- GCN + Dynamic Scene Fusion: A modified version of the Social-LSTM + Dynamic Scene Fusion model, in which the conventional social pooling mechanism is replaced by a GCN–based interaction module. This configuration explicitly models the relational dependencies among pedestrians and surrounding traffic participants through a graph structure, enabling structured message passing and context aggregation. The model preserves the use of dynamic scene features extracted from the VGG19 network, which are concatenated with trajectory embeddings to provide comprehensive environmental context. Although this model achieves higher prediction accuracy than Social-LSTM + Dynamic Scene Fusion due to its improved representation of inter-agent relationships, the use of VGG19-based panoramic scene feature extraction introduces considerable computational overhead, leading to longer training times. These results motivate the design of the proposed V-PTP-IC framework, which replaces heavy CNN-based scene encoding with a lightweight representation to achieve a more efficient balance between accuracy and computational cost.

As shown in Table 2 and Table 3, the proposed V-PTP-IC framework consistently outperforms all baseline models across both datasets, though the absolute performance on PIE is slightly lower than that on JAAD due to the inherent differences in dataset characteristics. Specifically, PIE focuses more on pedestrian intent and interactions with ego-vehicles rather than clear long-term trajectory annotation, resulting in fewer and less continuous trajectory samples. All experiments were conducted on a workstation equipped with an NVIDIA GeForce RTX 3050 GPU, ensuring a fair comparison of both predictive accuracy and computational efficiency. The inclusion of training time analysis further demonstrates the superior balance between performance and efficiency achieved by the proposed framework.

On the JAAD dataset, compared with the Vanilla LSTM, LSTM Encoder–Decoder, and Social-LSTM models, the proposed V-PTP-IC achieves relative improvements of 27.3%, 24.8%, and 20.0% in ADE, and 33.9%, 32.3%, and 28.5% in FDE, respectively. These results underscore the effectiveness of integrating dynamic scene features with the traffic object interaction graph network to enhance the accuracy of pedestrian trajectory prediction. Furthermore, compared with the Social-LSTM + Dynamic Scene Fusion model, V-PTP-IC attains an additional 6.4% and 10.7% improvement in ADE and FDE, respectively, indicating that the graph-based relational reasoning and lightweight feature extraction in V-PTP-IC provide a more comprehensive understanding of pedestrian motion within the surrounding traffic environment. Although the prediction accuracy improves significantly, V-PTP-IC exhibits a slight increase in training time (a 7.4% increase compared with the baseline), which is attributed to the additional computational overhead introduced by the extraction of traffic scene information.

On the PIE dataset, all models yield higher error values than those on JAAD due to increased motion complexity and the limited clarity of pedestrian trajectories. Nevertheless, V-PTP-IC consistently maintains superior performance, achieving 6.1% and 10.3% relative improvements in ADE and FDE, respectively, over the Social-LSTM + Dynamic Scene Fusion model. These consistent improvements across datasets confirm that the proposed framework generalizes well under varying levels of scene variability and spatio-temporal complexity.

As shown in Table 4, V-PTP-IC achieves the lowest ADE (0.0596) and FDE (0.1017) on the JAAD dataset with only 235.8K parameters and 1.38M FLOPs per trajectory. Compared with Social-LSTM, our model reduces ADE by 20.0% and FDE by 28.4% while incurring merely 58% more parameters and 23% additional FLOPs. Against the tiny Transformer that uses 28% more parameters (302.1K) and 67% higher computational cost (2.31M FLOPs), V-PTP-IC delivers 19.2% lower ADE and 27.3% lower FDE, demonstrating significantly superior accuracy-efficiency tradeoff. These results indicate that V-PTP-IC leverages moderate parameter and computation budgets to push the Pareto frontier of pedestrian trajectory prediction, achieving state-of-the-art accuracy without excessive complexity overhead.

### 4.5. Qualitative Evaluation

#### 4.5.1. Trajectory Stabilization Visualization

**Figure 2:**
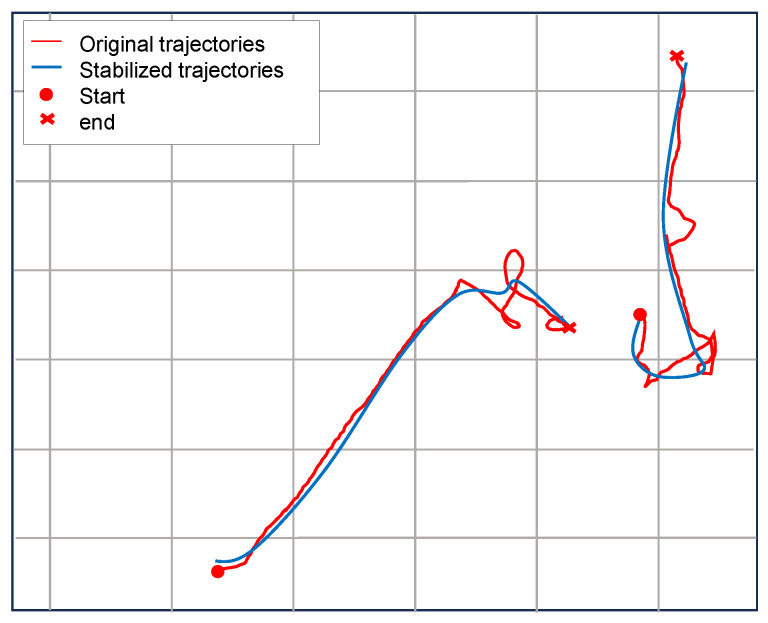
Comparison between original (red) and stabilized (blue) trajectories for the same pedestrian. SIFT-based stabilization substantially reduces high-frequency jitter and corrects discontinuities caused by detection errors and camera motion.

Figure 2 illustrates the qualitative comparison between raw and stabilized pedestrian trajectories. The original trajectory exhibits noticeable high-frequency oscillations and discontinuous jumps caused by ego-motion, detection noise, and temporary tracking failures. After applying SIFT-based stabilization, these distortions are substantially reduced, resulting in smoother and more temporally coherent trajectories. The static keypoints extracted from background structures serve as reliable geometric anchors, compensating for frame-to-frame motion drift induced by the moving camera. Consequently, the stabilized trajectories better align with the physical motion continuity of pedestrians and provide a more consistent representation for downstream modules. This improvement not only enhances visual interpretability but also leads to measurable gains in prediction stability, as smoother and spatially consistent inputs facilitate more accurate learning of spatio-temporal dependencies in subsequent interaction and trajectory prediction stages.

**Figure 3:**
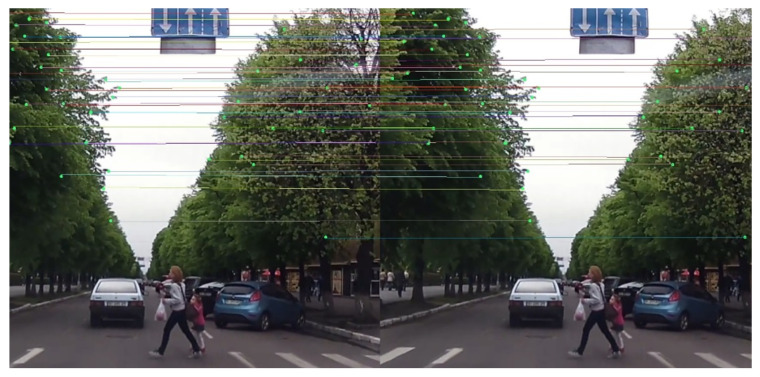
Static background keypoint matching between consecutive frames. These correspondences enable accurate estimation of global camera motion for trajectory stabilization.

Figure 3 presents the visualization of static background keypoint correspondences between consecutive video frames. The left and right images show matched SIFT keypoints connected by colored lines, representing stable background features such as trees, buildings, and road signs. These correspondences form the geometric foundation for estimating global camera motion via affine transformation. By leveraging the motion of these static anchors, the system effectively separates ego-motion from object motion, compensating for frame-to-frame displacement caused by vehicle movement. As a result, the global motion field derived from these keypoints directly enables accurate trajectory stabilization, suppressing translation and rotational artifacts that would otherwise distort pedestrian motion paths. This process ensures that the trajectory prediction modules receive spatially coherent inputs, thereby improving both trajectory smoothness and physical realism in dynamic driving scenes.

#### 4.5.2. Visualization of Trajectory Prediction

**Figure 4:**
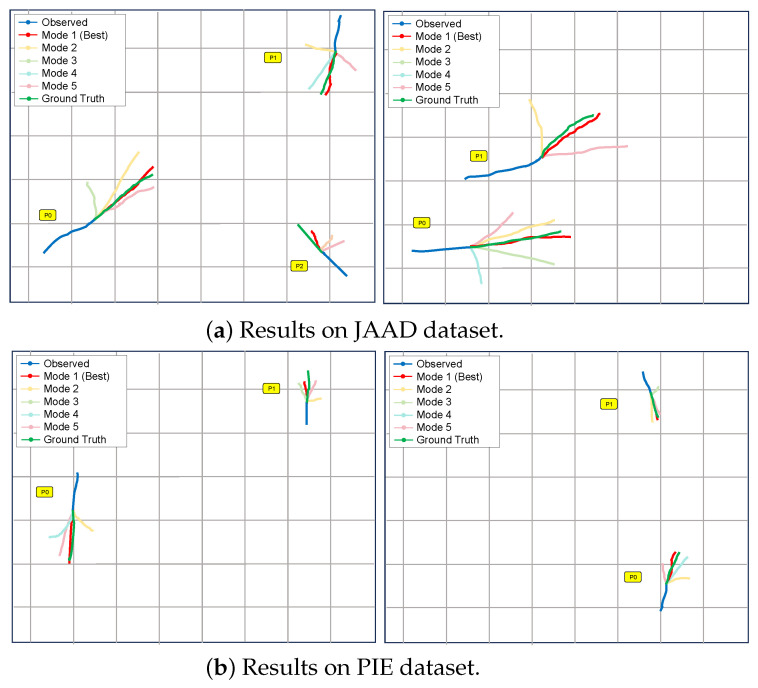
Visualization results of the proposed multimodal trajectory prediction, where blue indicates observed past trajectories, green denotes the ground truth, red represents the predicted most probable mode, and lighter curves correspond to alternative modes (2–5).

To further demonstrate the predictive performance and interpretability of the proposed framework, qualitative trajectory visualization experiments were conducted on both the JAAD and PIE datasets, as shown in Figure 4. The blue curves represent the observed historical trajectories, the green lines denote the ground-truth future paths, the red curves indicate the predicted most probable mode (Mode 1), and the lighter-colored curves (Modes 2–5) correspond to alternative plausible predictions generated by the multimodal decoder.

The visualization results demonstrate the advantages of our model: firstly, the model successfully captures the multimodal characteristics of pedestrian motion, generating diverse but physically reasonable future trajectories that cover different potential walking directions and speed curves. Secondly, the best prediction mode (Mode 1, red) shows strong consistency with the actual ground trajectory, which validates the effectiveness of our time smoothing constraint. However, visualization also reveals some limitations. In some complex intersecting scenarios, alternative modes (modes 2–5) occasionally produce trajectories that deviate significantly from the reasonable walking mode, indicating that there is still room for improvement in integrating stronger contextual understanding of the scene.

## 5. Conclusions

Our research proposes a novel end-to-end vehicle-centered framework, V-PTP-IC, for pedestrian trajectory prediction from a vehicle-mounted perspective, which jointly models stable pedestrian trajectories, scene semantics, and social interactions in driving environments. However, pedestrian trajectory prediction from a vehicle-mounted perspective encounters significant challenges, including trajectory jitter induced by camera ego-motion; frequent occlusions from dynamic viewpoints; and the lack of unified modeling for motion stabilization, multi-modal scene cues, and fine-grained multi-agent dependencies in uncertain real-world scenarios. To address these issues, V-PTP-IC integrates SORT for robust tracklet augmentation, a SIFT-based static keypoint matching strategy for compensating motion inconsistencies, capturing spatial regularities in dynamic scenes, and encoding behavioral interactions among traffic participants, thereby achieving robust trajectory estimation and generating geometrically accurate and socially compliant future paths. Furthermore, we mitigate the computational overhead and performance gaps in existing static- or global-view methods by leveraging lightweight scene features and attention-based fusion, enabling efficient processing of interaction dependencies without sacrificing prediction fidelity. Experimental results on the JAAD and PIE datasets indicate that V-PTP-IC outperforms baselines, with ADE reduced by 27.23% and 25.73% and FDE reduced by 33.88% and 32.85%, respectively, while maintaining low-latency inference suitable for real-time deployment.

From the perspective of vehicle-mounted observation, V-PTP-IC exhibits strong applicability for pedestrian trajectory prediction on traffic roads. However, precise trajectory prediction from a vehicle-mounted perspective remains challenging under adverse conditions such as low-light or occlusive weather, as well as for long-horizon forecasting in highly dynamic environments with limited temporal context. Future work will investigate multi-sensor fusion and Transformer-based sequential modeling to enhance environmental robustness and extend the temporal receptive field, alongside architecture optimizations for edge deployment to enable simultaneous multi-pedestrian predictions. These enhancements will further facilitate the integration of V-PTP-IC into safety-critical autonomous driving technologies.

## Figures and Tables

**Table 1:** Ablation study results on the JAAD dataset. Lower is better for ADE/FDE/Smoothness. Bold values indicate the best performance.

Configuration	ADE	FDE	Smoothness
Benchmark	0.1503	0.2042	0.1326
Exp1 (+SORT)	0.1298	0.1917	0.1104
Exp2 (+SORT + SIFT)	0.0981	0.1739	0.0983
Exp3 (+SORT + SIFT + Depth)	0.0714	0.1315	0.0895
Exp4 (Full Model)	**0.0596**	**0.1017**	**0.0657**
Exp5 (w/o SIFT)	0.1076	0.1908	0.0967
Exp6 (w/o Depth)	0.0744	0.1379	0.0715
Exp7 (w/o Scene)	0.0860	0.1542	0.0848

**Table 2:** Performance comparison of different models on the JAAD test set. Bold values indicate the best performance.

Model	ADE	FDE	Training Time	Smoothness	Collision Rate
Vanilla LSTM [17]	0.0819	0.1538	1600.47	0.1009	–
LSTM-Encoder-Decoder [40]	0.0792	0.1502	1566.39	0.0987	–
Social-LSTM [17]	0.0745	0.1421	1459.34	0.0856	0.041
Transformer [41]	0.0738	0.1398	1470.21	0.0823	–
Social-LSTM + Dynamic Scene Fusion	0.0637	0.1140	1507.86	0.0686	0.027
GCN + Dynamic Scene Fusion	0.0618	0.1103	1581.92	0.0703	0.020
V-PTP-IC (Ours)	**0.0596**	**0.1017**	**1482.52**	**0.0657**	**0.019**

**Table 3:** Performance comparison of different models on the PIE test set. Bold values indicate the best performance.

Model	ADE	FDE	Training Time	Smoothness	Collision Rate
Vanilla LSTM [17]	0.1026	0.1927	1615.28	0.1182	–
LSTM-Encoder-Decoder [40]	0.0991	0.1863	1580.77	0.1149	–
Social-LSTM [17]	0.0927	0.1748	1496.54	0.1038	0.052
Transformer [41]	0.0916	0.1705	1512.83	0.0996	–
Social-LSTM + Dynamic Scene Fusion	0.0803	0.1442	1538.65	0.0851	0.038
GCN + Dynamic Scene Fusion	0.0787	0.1386	1602.14	0.0869	0.031
V-PTP-IC (Ours)	**0.0762**	**0.1294**	**1510.42**	**0.0835**	**0.028**

**Table 4:** Model complexity versus prediction accuracy on JAAD dataset (obs = 8, pred = 12, BS = 1). ↓ indicates lower is better.

Model	Params ↓	FLOPs ↓	ADE ↓	FDE ↓
Vanilla LSTM	67.8 K	0.43 M	0.0819	0.1538
LSTM Encoder-Decoder	134.2 K	0.86 M	0.0792	0.1502
Social-LSTM	148.7 K	1.12 M	0.0745	0.1421
Transformer (tiny)	302.1 K	2.31 M	0.0738	0.1398
V-PTP-IC (Ours)	235.8 K	1.38 M	0.0596	0.1017

## Data Availability

The data supporting the findings of this study are openly available in the JAAD (Joint Attention in Autonomous Driving) dataset at https://github.com/ykotseruba/JAAD (accessed on 20 November 2025). The dataset includes video sequences and annotations for pedestrian behavior analysis in autonomous driving scenarios.
